# A single poly-Si gate-all-around junctionless fin field-effect transistor for use in one-time programming nonvolatile memory

**DOI:** 10.1186/1556-276X-9-603

**Published:** 2014-11-06

**Authors:** Mu-Shih Yeh, Yung-Chun Wu, Kuan-Cheng Liu, Ming-Hsien Chung, Yi-Ruei Jhan, Min-Feng Hung, Lun-Chun Chen

**Affiliations:** 1 Department of Engineering and System Science, National Tsing Hua University, 101, Section 2, Kuang Fu Road, Hsinchu 30013, Taiwan

**Keywords:** Single poly-Si, Gate-all-around, Junctionless, Fin field-effect transistor, One-time programming, Nonvolatile memory, Three-dimensional, Flash memory

## Abstract

This work demonstrates a feasible single poly-Si gate-all-around (GAA) junctionless fin field-effect transistor (JL-FinFET) for use in one-time programming (OTP) nonvolatile memory (NVM) applications. The advantages of this device include the simplicity of its use and the ease with which it can be embedded in Si wafer, glass, and flexible substrates. This device exhibits excellent retention, with a memory window maintained 2 V after 10^4^ s. By extrapolation, 95% of the original charge can be stored for 10 years. In the future, this device will be applied to multi-layer Si ICs in fully functional systems on panels, active-matrix liquid-crystal displays, and three-dimensional (3D) stacked flash memory.

## Background

**Figure 1:**
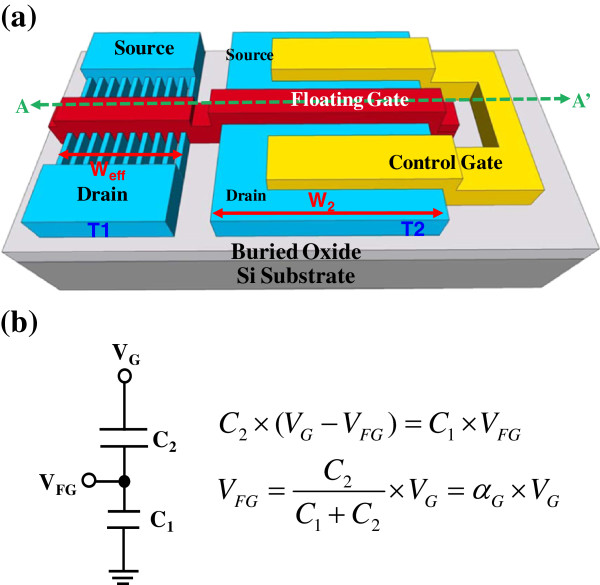
**Schematic and equivalent circuit of the single poly-Si JL-FinFET GAA NVM. (a)** Schematic of the single poly-Si JL-FinFET GAA NVM with ten NWs and **(b)** its equivalent circuit. Two transistors, T1 (NW channel) and T2 (wide channel), are connected by a floating gate (FG), and the source and drain of T2 are connected as the controlling gate (CG). The simplified calculation of the voltage in floating gate is appended in **(b)**.

Twin thin-film transistor (TFT) nonvolatile memory (NVM) [1,2] tunnel oxide and blocking oxide were formed simultaneously as a ‘single poly-Si layer’. The simple process flow allows a logic circuit to be easily embedded in this device, reducing process costs. The coupling ratio of the memory can be easily controlled by setting the ratio T1/T2 according to the formula. Figure 1b shows the equivalent circuit of this single poly-Si gate-all-around (GAA) junctionless fin field-effect transistor (JL-FinFET) NVM:

$$V_{FG}=\;\left[C_{2}/\;\left(C_{1}+C_{2}\right)\right]\;\times \;V_{G}=\;\left[W_{2}/\;\left(W_{\mathrm{eff}}+W_{2}\right)\right]\;\times \;V_{G}=\alpha_{G}\times V_{G}$$

**Figure 2:**
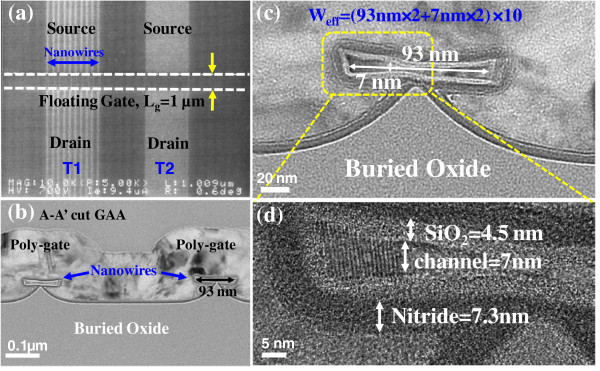
**SEM and TEM images of the single poly-Si JL-FinFET GAA NVM. (a)** The top-view SEM image of the active region of single poly-Si JL-FinFET GAA NVM with gate length (*L*_g_) = 0.1 μm. **(b)** The TEM image of cross-section of the single poly-Si JL-FinFET NVM with GAA-NWs. **(c)** The effective channel width (*W*_eff_) is 200 nm × 10 [(93 nm × 2 + 7 nm × 2) × 10)]. **(d)** The oxide/nitride layers are designed to be 4.5 nm thick/7.3 nm thick.

The single poly-Si GAA JL-FinFET NVM has a coupling ratio of 0.85, which is much larger than that of the conventional stacked memory. In programming, the electrons tunnel into T1 through the tunneling oxide. The tunneling oxide of nanowire (NW)-based NVM is surrounded by the gate electrode. To maximize the voltage drop in the tunnel oxide of T1, the gate capacitance of T2 (*C*_2_) must exceed the gate capacitance of T1 (*C*_1_). Hence, the NVM device with a high artificial gate coupling ratio (*α*_G_) exhibits a high program speed and can be operated at a low voltage. Noteworthily, the particular planar twin-TFT NVM structure enables the *C*_1_, *C*_2_, and *α*_G_ to be easily designed. The device was designed to have a coupling ratio of 0.85 by setting T1/T2 = 1 μm/6 μm (Figure 2a). The single poly-Si GAA JL-FinFET NVM can be easily incorporated into SOI CMOS technology without additional processing [3-5].

In this work, a JL channel [6-11] is introduced into the single poly-Si structure. The use of the JL channel enables the short channel effect (SCE) to be reduced and a simple implantation process to be used. The single poly-Si JL-FinFET NVM reduces the leakage current, according to Moore’s law, significantly reducing the development time and fabrication cost. JL NW devices with high doping concentrations in their channel and source/drain (S/D) regions have attracted much interest. The advantages of these devices over conventional inversion mode devices are (1) lack of need for an ultra-shallow S/D junction, which simplifies the fabrication process; (2) a low thermal budget, because of the elimination of the need for implant activation annealing after gate stack formation; and (3) concentration of the current of the JL device on the bulk of the semiconductor, which reduces the adverse effects of imperfect interfaces between the semiconductor and the insulator.

The channel is designed with a GAA structure to ensure that the gate can be effectively controlled [12,13]. GAA devices have an on/off ratio of 10^7^ and a subthreshold swing (SS) of 150 mV/decade, which can be easily obtained when the thickness of the JL channel is well controlled [14,15].

The one-time programming (OTP) memory device can be programmed at high speed; it exhibits excellent retention, with a memory window maintained 2 V after 10^4^ s. By extrapolation, 95% of the original charge can be stored for 10 years. Therefore, this work develops a high-performance single poly-Si GAA JL-FinFET NVM, which has high programming speed and excellent reliability.

## Methods

In this work, a single poly-Si JL-FinFET NVM with an oxide/nitride layer is fabricated. Figure 1a schematically depicts the single poly-Si JL-FinFET NVM with ten NWs and the device has GAA-NWs. The gate electrodes of two TFTs are connected to form the floating gate (FG), while the source and drain of the larger TFT (T2) are connected to form the control gate.

The aforementioned devices were fabricated by initially growing a 400-nm-thick thermal oxide layer on 6-in. silicon wafers as substrates. A thin 50-nm-thick undoped amorphous-Si (a-Si) layer was then deposited by low-pressure chemical vapor deposition (LPCVD) at 550°C. The deposited a-Si layer was then solid-phase crystallized (SPC) at 600°C for 24 h in nitrogen ambient. A 2 × 10^14^ cm^−2^ dose of phosphorus was implanted and the device was then annealed to form the n-type active layer. The active device was patterned by electron beam (e-beam) direct writing and transferred by reactive ion etching (RIE). The device was dipped in hydrogen fluoride (HF) solution for 120 s to suspend the NWs in midair.

To enable the JL device to be controlled, the channel must be trimmed in the thickness direction. Therefore, the naked NWs can form by thermal oxidation which grew the 24-nm oxide layer and then dipped in HF for 100 s to remove the first sacrificial oxide layer. The dipped HF condition can keep the buried oxide sustain NWs (Figure 2c). Secondly, thermal oxidation is performed to produce a tunnel oxide layer with a thickness of 3 nm. Then, LPCVD is conducted to deposit a 7-nm-thick nitride layer as storage layer. The gate regions are formed by *in situ* doping with phosphorus ions to form the n-type poly gate. The channel of the device thus formed has a GAA structure.

On such devices, a 200-nm-thick TEOS oxide layer was deposited as the passivation layer by LPCVD. Next, the contact holes were defined and a 300-nm-thick Al-Si-Cu metallization was performed. Finally, the device was sintered at 400°C in nitrogen ambient for 30 min.

## Results and discussion

**Figure 3:**
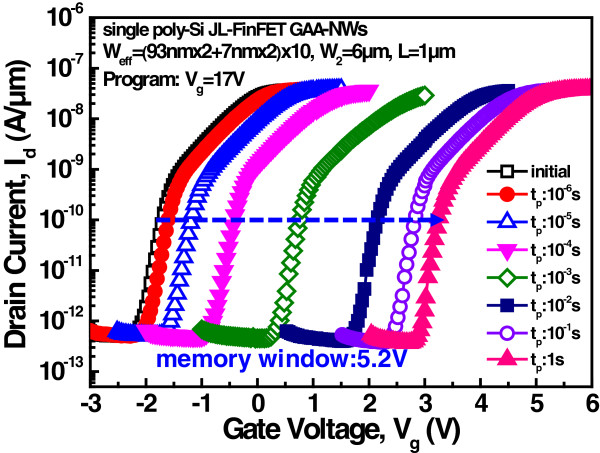
**Program characteristics of the single poly-Si JL-FinFET GAA NVM.** With F-N programming, the memory window opens to 5.2 V for 1 s. Erase is invalid.

**Figure 4:**
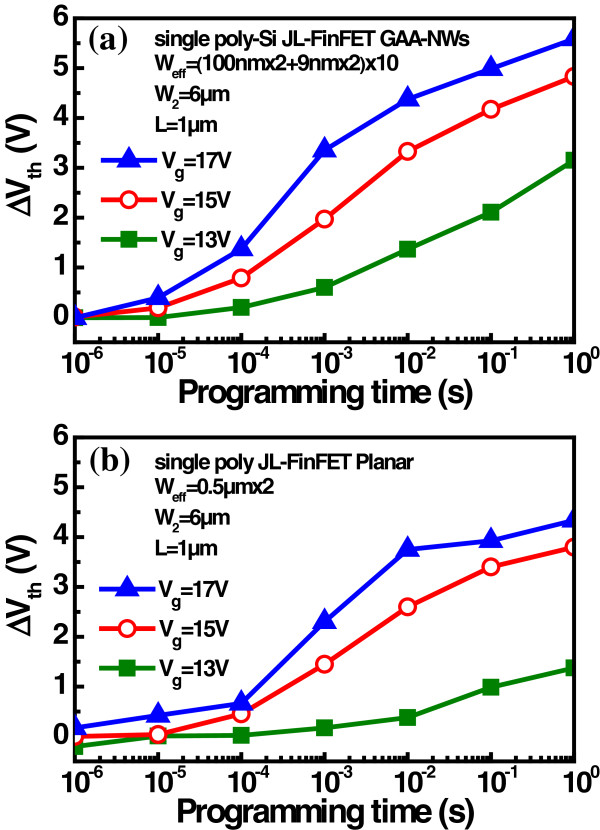
**Programming speed characteristics of the single poly-Si JL-FinFET NVM.** Program speed characteristic of **(a)** JL-FinFET with GAA-NWs and **(b)** JL-FinFET with planar structures, respectively.

Figure 2a shows the top-view scanning electron microscopic (SEM) image of the active region of a single poly-Si JL-FinFET GAA NVM with gate length (*L*_g_) = 0.1 μm. Figure 2b presents a cross-sectional transmission electron microscopy (TEM) image of a single poly-Si JL-FinFET NVM with GAA-NWs. Figure 2c,d shows the effective channel width is 200 nm × 10 [(93 nm × 2 + 7 nm × 2) × 10)]. The oxide/nitride layers are designed to be 4.5 nm thick/7.3 nm thick as well as the entire device.To operate the memory, the device is programmed by Fowler-Nordheim (F-N) tunneling by applying 17 V, as presented in Figure 3. The memory window is opened to 5.2 V for 1 s. This device cannot be erased when a negative bias is applied, perhaps because of the high carrier density in the channel and the fact that the channel does not have sufficient holes to be able to erase. Figure 4a,b displays the programming speeds of the device GAA-NW and the planar device, respectively. The GAA-NW device has a higher programming speed than the planar device because of the field enhancement effect at corners of the oxide layer.

Recently, the embedded NVM has been extensively applied to different systems [16]. A device that can only be programmed once, which is called a ‘one-time programmable device’ , plays an important role in CMOS technology. OTP has always attracted much interest because of the ease of the process flow and its high performance, which reduce the cost of the process.

**Figure 5:**
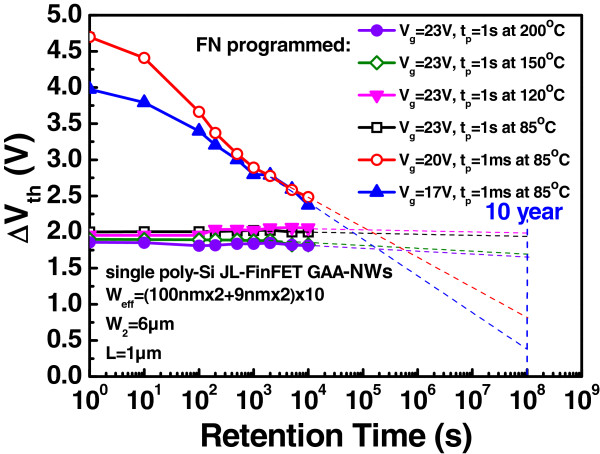
**Retention characteristics.** Retention characteristics of the JL-FinFET GAA-NW device using different voltages and temperatures.

An OTP device [17,18] cannot be repeatedly operated; it is used for its good retention. Figure 5 shows the retention testing of such a device with the GAA-NW structure programmed using the F-N operation. When a voltage of more than 23 V was applied for 1 s, independently of the temperature (from 85°C to 200°C), the device exhibited perfect retention. In a retention test, at least 95% of the original charge remained after 10 years. This excellent retention makes the device a good OTP device. The device exhibits perfect retention when large pulses are applied one at a time separately and consecutively, pushing electrons into the FG region or into deep traps in the silicon nitride.

**Figure 6:**
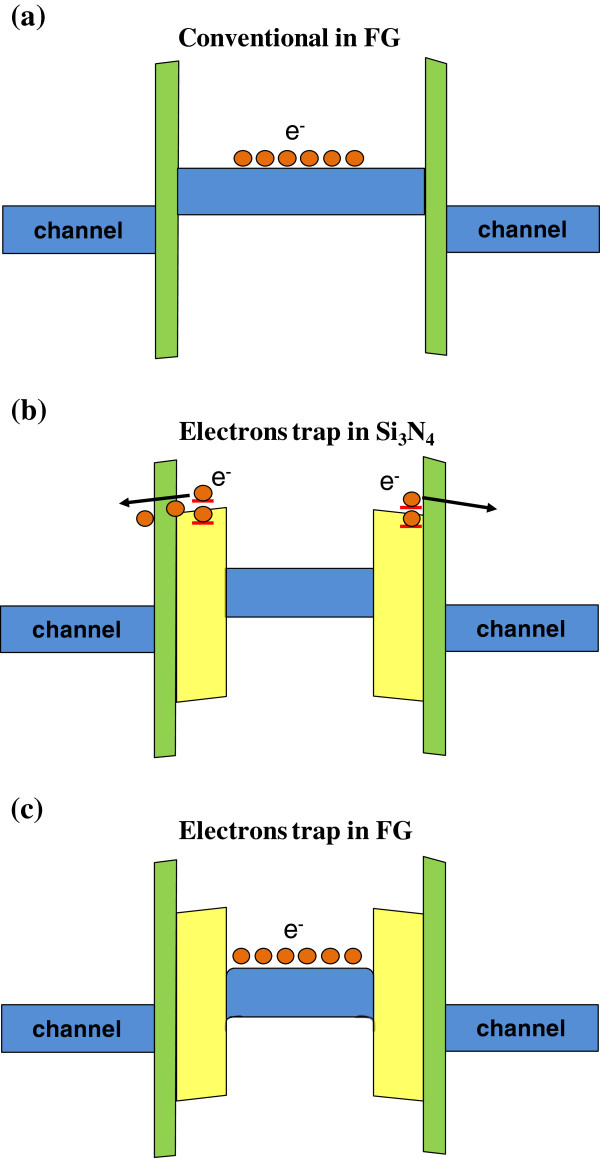
**Energy band diagram in the retention state of the single poly-Si JL-FinFET GAA NVM. (a)** The conventional FG memory devices. Band diagram of retention state for operation voltage **(b)** lower than 23 V for 1 s and **(c)** larger than 23 V for 1 s.

Figure 6 presents the energy band diagram in the retention state; Figure 6a shows the conventional FG memory devices. After a program cycle, some of the electrons will be stored in the interface trap between the channel and oxide, and these stored electrons induce the leakage current. Since the leaking possibility of the electrons is increasing, conventional FG memory devices are resulting in poor reliability and retention. Figure 6b shows the conditions for programming the device using voltages of less than 23 V. Some of the charges are stored in the shallow trapping layer of nitride or at the interface between the oxide and the nitride layer. Under high-temperature conditions, these charges may leak out by trap-assisted tunneling [19,20] or direct tunneling [21] through the oxide, resulting in poor retention. In contrast, when the programming voltage is higher than 23 V for 1 s, charges that are trapped in the nitride may undergo trap-assisted tunneling, and all such charges are pushed into the FG region as shown in Figure 6c, because the nitride layer has many trap sites and the electron will encounter a high potential barrier when it is stored in the poly-Si region. Hence, the device in this work is favorable for OTP applications and exhibits excellent retention and reliability.

## Conclusions

This work demonstrates the potential of the single poly-Si JL-FinFET GAA NVM for use in OTP operations and its easy embedding into logic circuits. The device can be programmed by FN tunneling but not easily erased, owing to the high dopant concentration. The GAA structure provides good control ability. The device exhibits excellent retention and the memory window can be maintained at 2 V after 10^8^ s, with at least 95% of the original charge stored for more than 10 years. At 200°C, in a high-temperature retention test, the device exhibits better retention than at low temperature. Measurements demonstrate that the device has a lower fabrication cost and greater scalability than the conventional OTP memory. Therefore, the single poly-Si JL-FinFET GAA NVM is an effective embedded NVM for advanced logic technologies.

## Competing interests

The authors declare that they have no competing interests.

## Authors’ contributions

M-SY and K-CL carried out the device mask layout, modulated the couple ratio of the device, handled the experiment, and drafted the manuscript. M-HC measured the characteristics of the device. Y-RJ and L-CC gave some physical explanation to this work. M-FH participated in the design and coordination of the study. Y-CW conceived the low-temperature deposition of the single poly-Si JL-FinFET GAA NVM idea and their exploitation into OTP devices. He supervised the work and reviewed the manuscript. All authors read and approved the final manuscript.
